# Fever Is Mediated by Conversion of Endocannabinoid 2-Arachidonoylglycerol to Prostaglandin E_2_

**DOI:** 10.1371/journal.pone.0133663

**Published:** 2015-07-21

**Authors:** Yoshihiro Kita, Kenij Yoshida, Suzumi M. Tokuoka, Fumie Hamano, Maya Yamazaki, Kenji Sakimura, Masanobu Kano, Takao Shimizu

**Affiliations:** 1 Department of Lipidomics, Graduate School of Medicine, The University of Tokyo, Bunkyo-ku, Tokyo, Japan; 2 Life Sciences Core Facility, Graduate School of Medicine, The University of Tokyo, Bunkyo-ku, Tokyo, Japan; 3 Department of Cellular Neurobiology, Brain Research Institute, Niigata University, Niigata, Niigata, Japan; 4 Department of Neurophysiology, Graduate School of Medicine, The University of Tokyo, Bunkyo-ku, Tokyo, Japan; 5 Department of Lipid Signaling, Research Institute, National Center for Global Health and Medicine, Shinjuku-ku, Tokyo, Japan; St. Joseph's Hospital and Medical Center, UNITED STATES

## Abstract

Fever is a common response to inflammation and infection. The mechanism involves prostaglandin E_2_ (PGE_2_)-EP3 receptor signaling in the hypothalamus, which raises the set point of hypothalamic thermostat for body temperature, but the lipid metabolic pathway for pyretic PGE_2_ production remains unknown. To reveal the molecular basis of fever initiation, we examined lipopolysaccharides (LPS)-induced fever model in monoacylglycerol lipase (MGL)-deficient (*Mgll* ^−/−^) mice, CB1 receptor-MGL compound-deficient (*Cnr1* ^−/−^ *Mgll* ^−/−^) mice, cytosolic phospholipase A_2_α (cPLA_2_α)-deficient (*Pla2g4a* ^−/−^) mice, and diacylglycerol lipase α (DGLα)-deficient (*Dagla* ^−/−^) mice. Febrile reactions were abolished in *Mgll* ^−/−^ and *Cnr1* ^−/−^ *Mgll* ^−/−^ mice, whereas *Cnr1* ^−/−^ *Mgll* ^+/+^, *Pla2g4a* ^−/−^ and *Dagla* ^−/−^ mice responded normally, demonstrating that MGL is a critical enzyme for fever, which functions independently of endocannabinoid signals. Intracerebroventricular administration of PGE_2_ caused fever similarly in *Mgll* ^−/−^ and wild-type control mice, suggesting a lack of pyretic PGE_2_ production in *Mgll* ^−/−^ hypothalamus, which was confirmed by lipidomics analysis. Normal blood cytokine responses after LPS administration suggested that MGL-deficiency does not affect pyretic cytokine productions. Diurnal body temperature profiles were normal in *Mgll* ^−/−^ mice, demonstrating that MGL is unrelated to physiological thermoregulation. In conclusion, MGL-dependent hydrolysis of endocannabinoid 2-arachidonoylglycerol is necessary for pyretic PGE_2_ production in the hypothalamus.

## Introduction

Fever is one of the typical responses to inflammation and infection. It is thought to be an adaptive defense mechanism, as higher body temperature prevents propagation of infectious microorganisms and activates the host's immune system [1]. In homeotherms including mammals, body temperature is regulated by the control of heat production and heat loss to adjust it to a ‘set point’, typically set at around 37°C in mammals [2]. Fever is explained as a ‘resetting’ of the set point to a higher value, which raises the body temperature thresholds for heat production and heat loss mechanisms, causing increased muscle tone, shivering and peripheral vasoconstrictions until the body temperature reaches the new set point [2].

Upon infection of viruses or bacteria, host’s immune cells produce pyretic cytokines including TNFα, IL-1β, and IL-6 [3]. These cytokines via circulation stimulate a production of prostaglandin E_2_ (PGE_2_), an arachidonic acid (AA)-derived lipid mediator, in the hypothalamus. PGE_2_ is considered to be a critical mediator of fever, as genetic ablations of genes for enzymes involved in biosynthesis of PGE_2_, such as cyclooxygenase-2 (COX-2, encoded by *Ptgs2*) and microsomal prostaglandin E synthase-1 (mPGES-1, encoded by *Ptges*), abolish febrile reactions in mice [4, 5]. Among 4 known PGE_2_ receptor subtypes [6], EP3 receptors (EP3R, encoded by *Ptger3*) mediate fever, as global *Ptger3*-knockout [7] and conditional *Ptger3*-knockout in the medial preoptic nucleus of the hypothalamus [8] prevent fever responses in mice.

A number of previous studies have demonstrated that AA, a precursor for PGE_2_, is liberated from AA-containing phospholipids by the action of phospholipases A_2_ (PLA_2_s) [9]. Among PLA_2_s, cytosolic PLA_2_α (cPLA_2_α, group IVA PLA_2_) encoded by *Pla2g4a* gene, has been known as a crucial enzyme for eicosanoid production in inflammatory cells [9]. Although it has been suggested that cPLA_2_α is involved in PGE_2_ production during fever [10], this has not been clearly demonstrated. An alternative AA-producing pathway from 2-arachidonoylglycerol (2-AG), an endocannabinoid that functions in the central nervous system through activation of CB1 cannabinoid receptors (CB1R, encoded by *Cnr1* gene), was first documented in platelets [11, 12]. A recent study demonstrated that deletion of *Mgll*, a gene that encodes monoacylglycerol lipase (MGL) which hydrolyzes 2-AG to AA and glycerol, results in accumulation of 2-AG and consequent reductions of AA and eicosanoid levels in the mouse brain [13], suggesting an importance of this pathway.

Here, we address the biosynthetic pathway for pyretic PGE_2_ in the hypothalamus. Using null mutant mice, we found that lipopolysaccharides (LPS)-induced fever depends on MGL but not on cPLA_2_α. *Mgll* ^−/−^ mice showed normal cytokine responses after LPS administration, but had reduced hypothalamic PGE_2_ levels. When injected intracerebroventricularly (ICV), PGE_2_ caused fever in *Mgll* ^−/−^ mice. These data demonstrate that MGL-dependent hypothalamic PGE_2_ is required for a febrile response. We also show that diurnal core body temperature changes are normal in MGL-KO mice, demonstrating that MGL is not critical in physiological thermoregulations.

## Materials and Methods

### Reagents

Lipopolysaccharides (LPS, from *Escherichia Coli*, serotype 0111:B4) were purchased from Sigma-Aldrich (St. Louis, MO). Organic solvents for liquid chromatography and lipid extraction (methanol, acetonitrile, chloroform) and modifiers (formic acid and ammonium bicarbonate) were purchased from Wako (Osaka, Japan). Phospholipids and eicosanoids were purchased from Avanti Polar Lipids (Alabaster, AL) and Cayman Chemical (Ann Arbor, MI), respectively. Fatty acids and fatty acid methyl esters were purchased from Wako and Sigma-Aldrich, respectively.

### Animals

MGL-deficient (*Mgll* ^−/−^) mice [14], DGLα-deficient (*Dagla* ^−/−^) mice [15], CB1R-MGL compound-deficient (*Cnr1* ^−/−^ *Mgll* ^−/−^) mice, cytosolic phospholipase A_2_α-deficient (*Pla2g4a* ^−/−^) mice [16], and their control mice as specified in each experiment, were prepared by mating respective heterozygous mice. Female mice were used for all the studies. All mouse strains had a C57BL/6 genetic background and were maintained in a specific-pathogen-free facility (ambient temperature 23°C, light-dark cycle with lights on from 0700 to 2000 h), and fed *ad libitum* with a standard laboratory diet (MF; Oriental Yeast, Tokyo, Japan) and water.

### LPS-induced fever model

Adult female mice (8–10 wk-old) were isolated and habituated to an ambient temperature of 30°C before the experiment. LPS (20 μg dissolved in 100 μL saline) or saline was injected intraperitoneally, and core body temperatures were monitored every 30 min using a RET-3 rectal probe (AD instruments, Dunedin, New Zealand) and a MR2041 thermo-logger (Chino, Tokyo, Japan). Mice were habituated to the insertion of rectal probe 3–4 h before LPS injection. We observed relatively unstable body temperature readings at 30 min, 60 min, and 90 min timepoints, which can be attributed to injection and/or restraint stress, and therefore, we did not evaluate these datapoints. Fever typically manifested ~2h after LPS injection.

### ICV administration of PGE_2_

For intracerebroventricular injection, mice were anesthetized with isoflurane using a Univentor 400 anesthesia unit (Univentor, Zejtun, Malta). Using a Hamilton Gastight micro syringe (Reno, NV) with a 27-gauge needle (Terumo, Tokyo, Japan), PGE_2_ (4 nmol in 2 μL of saline) or equal volume of saline was injected to the left lateral ventricle (coordinates: 1 mm lateral and 0.5 mm caudal to bregma, depth 2.5 mm). After injection, mice were recovered from anesthesia and core body temperatures were monitored every 15 min using a rectal probe. Ambient temperature was set at 25°C.

### Measurement of diurnal core body temperature changes

A miniature temperature data logger device was prepared according to the method reported previously [17], with modifications. In brief, the circuit board of DS1921H-F5# iButton device (Maxim Integrated Products, San-Jose, CA) was removed, reassembled with a new lithium coin battery (CR1216, Panasonic, Osaka, Japan), programmed to record the temperature every 5 min, and then potted with paraffin (m.p. 68–70°C, Wako) to make it water-proof. Mice were intra-abdominally implanted with the thermo-loggers and maintained under a light-dark cycle with lights on from 0800 to 2000 h. Ambient temperature was set at 25°C. Ten days after surgery, the loggers were removed from mice and incubated at 37°C for 30 min for post-calibration purpose. The data were retrieved using OneWireViewer software (Maxim). Data points for 3 consecutive light-dark cycles (day 8–day 10 after surgery) were used to determine the diurnal core body temperature changes for each mouse.

### Lipid analysis

Hypothalamus block and liver samples were collected from mice 2 h after intraperitoneal injection of LPS or saline. Lipids were extracted from tissues stored frozen under liquid nitrogen, by methanol (for eicosanoids), acetonitrile (for 2-AG and AEA), or chloroform-methanol (1:1, for total fatty acids). Eicosanoids, 2-AG, and AEA were measured using liquid chromatography mass spectrometry (LC-MS) as previously described [15, 18]. Total fatty acids were analyzed by a gas chromatograph with a flame ionization detector (GC-2010 Plus, Shimadzu, Kyoto, Japan) using a Supelco SP-2560 column (100 m × 0.25 mm I.D., 0.20 μm, Sigma-Aldrich). Total lipid samples were spiked with C21:0 fatty acid internal standard, and derivatized to fatty acid methyl esters (FAMEs) using a FAME derivatization and purification kit (Nacalai Tesque, Kyoto, Japan). The samples were quantified using peak areas of known amounts of FAMEs and C21:0 FAME. Phospholipid profiling analysis was conducted with a Nexera liquid chromatograph connected to an LCMS-8040 triple quadrupole mass spectrometer with electrospray-ionization source (Shimadzu).

### Serum cytokines

Serum samples were collected from mice 2 h after intraperitoneal injection of LPS or saline. TNFα, IL-1β, and IL-6 were determined by enzyme-linked immunosorbent assay kits (Thermo Scientific, Waltham, MA).

### Statistics

GraphPad Prism 5 (GraphPad Software, San Diego, CA) was used for statistical calculations. *P*-values < 0.05 were considered statistically significant.

### Ethics statement

All procedures in the present study were conducted in accordance with the Guidelines for Animal Care of The University of Tokyo, approved by the animal experimentation committee of Faculty of Medicine, The University of Tokyo (approval no. M-P12-124).

## Results

### MGL mediates LPS-induced febrile response

**Fig 1:**
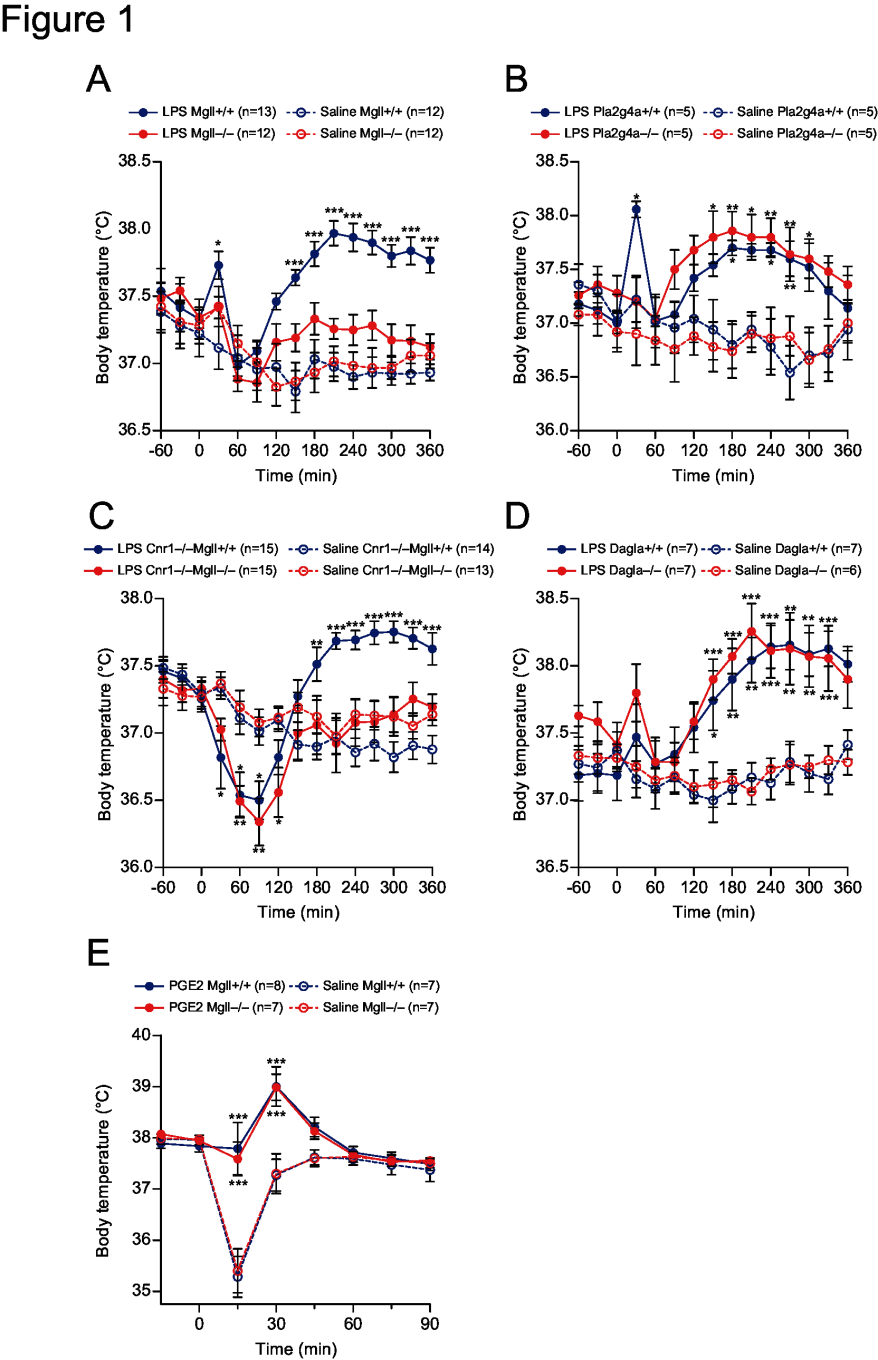
LPS- and PGE_2_-induced febrile responses. (A-D) MGL-knockout (*Mgll* ^−/−^) mice (A), cPLA_**2**_α-knockout (*Pla2g4a* ^−/−^) mice (B), CB1R-MGL compound-knockout (*Cnr1* ^−/−^ *Mgll* ^−/−^) mice (C), DGL-knockout (*Dagla* ^−/−^) mice (D), and their respective WT control mice were intraperitoneally injected with 20 μg of LPS or saline (n = 5–15 as indicated on the panels). Core body temperatures were monitored by insertion of a rectal probe every 30 min. Mice were kept at thermo-neutral conditions (30°C). (E) *Mgll* ^+/+^ and *Mgll* ^−/−^ mice were intracerebroventricularly injected with 4 nmol of PGE_**2**_ or saline (n = 7–8 for each group). Core body temperatures were monitored by a rectal probe every 15 min. Mice were kept at 23°C. Data are expressed as means ± SEM. *p<0.05, ** *p*< 0.01, ***p<0.001 vs. saline-treated wild-type controls, by Bonferroni post-test after two-way repeated measures ANOVA.

We first examined LPS-induced fever in mice lacking MGL or cPLA_2_α to identify which enzyme mediates PGE_2_ production during fever. Bacterial endotoxin LPS activates TLR4 receptors expressed on immune cells, causing various inflammatory responses including immediate release of proinflammatory cytokines [19]. Intraperitoneal administration of LPS (20 μg dissolved in 100 μL saline; from *Escherichia Coli*, serotype 0111:B4) caused a transient increase in core body temperature, which was typically observed in wild-type mice 2–6 h after LPS administration (Fig 1*A* and 1*B*). The febrile responses were largely attenuated in *Mgll* ^−/−^ mice (Fig 1*A*), while remaining unchanged in *Pla2g4a* ^−/−^ mice (Fig 1*B*). Ablation of MGL is reported to increase 2-AG levels, which reportedly results in downregulation and desensitization of CB1R in the brain [20]. To examine if the attenuated febrile responses in *Mgll* ^−/−^ mice were due to altered CB1R signals, compound knockout mice for *Cnr1* and *Mgll* genes (*Cnr1* ^−/−^ *Mgll* ^−/−^) were examined. LPS-induced fever was blocked in *Cnr1* ^−/−^ *Mgll* ^−/−^ mice, while control *Cnr1* ^−/−^ *Mgll* ^+/+^ mice showed normal febrile responses (Fig 1*C*). These results demonstrated that the impaired febrile responses in *Mgll* ^−/−^ mice are not due to altered CB1R signaling in these mice. We also examined the possible involvement of diacylglycerol lipase α (DGLα), a major 2-AG-producing enzyme in the brain [15]. LPS-induced febrile responses in diacylglycerol lipase α-deficient (*Dagla* ^−/−^) mice were normal (Fig 1*D*), suggesting that this enzyme is not involved in fever mechanism.

### ICV administration of PGE_2_ elicits fever in MGL-deficient mice

We next performed ICV administration of PGE_2_ (4 nmol in 2 μL of saline) to *Mgll* ^−/−^ and control WT mice. PGE_2_ caused transient fever that reached a peak around 30 min after its administration similarly in *Mgll* ^−/−^ and control WT mice (Fig 1*E*). Vehicle injection (2 μL of saline) caused transient hypothermia in the early (~15 min) phase, which was not evident in PGE_2_-injected mice and was similarly observed in both genotypes (Fig 1*E*). The result demonstrated that fever mechanisms downstream of PGE_2_ are intact in *Mgll* ^−/−^ mice.

### Systemic cytokine response to LPS administration is normal in MGL-deficient mice

**Fig 2:**
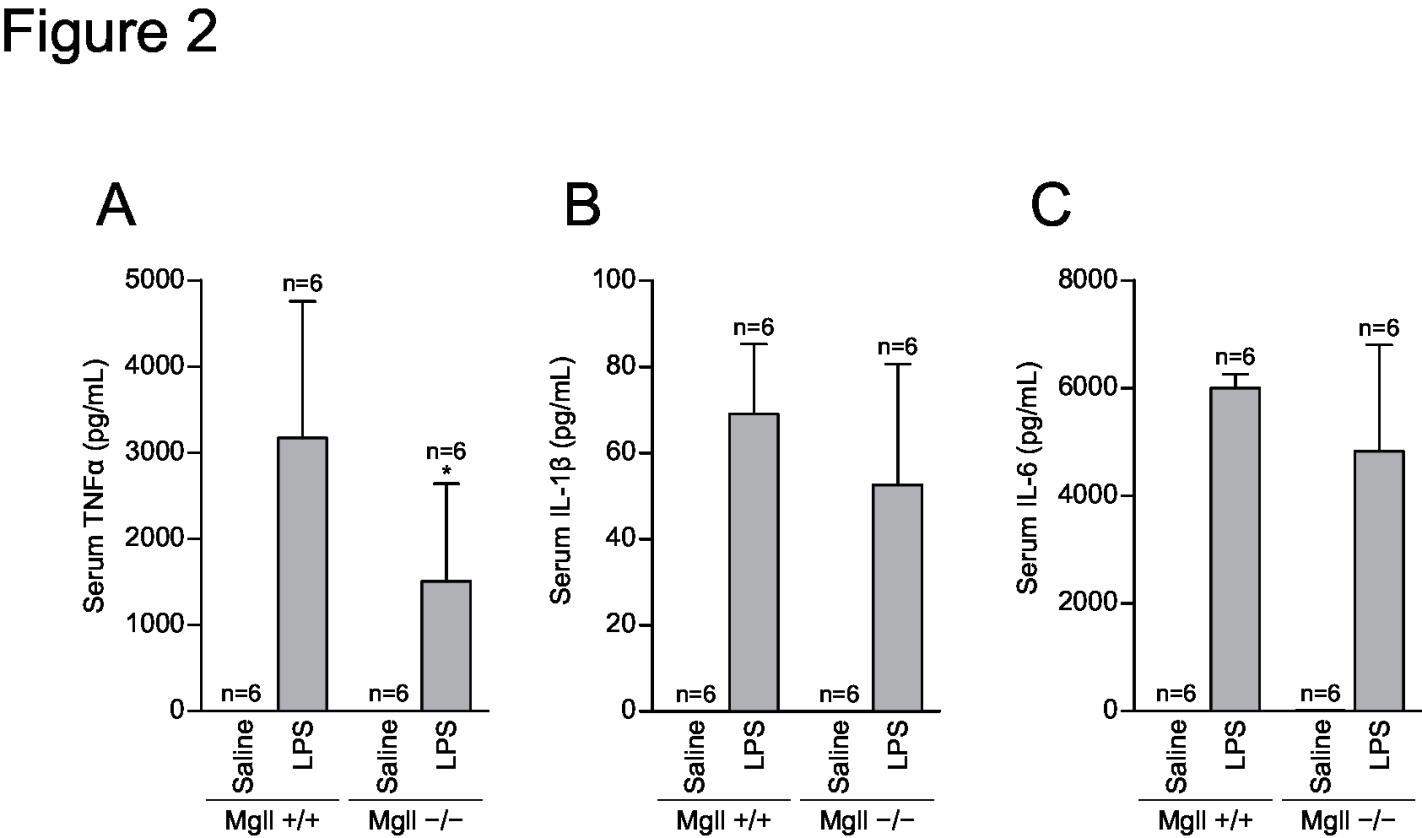
Serum cytokines after LPS administration. *Mgll* ^+/+^ and *Mgll* ^−/−^ mice (n = 6 for each group) were intraperitoneally injected with 20 μg of LPS or saline, and serum were collected 2 h after injection. Serum levels of TNFα (A), IL-1β (B), and IL-6 (C) were determined by enzyme-linked immunosorbent assay. Data are expressed as mean ± SD. *p<0.05 vs. *Mgll* ^+/+^, by Bonferroni post-test after two-way ANOVA.

A previous report demonstrated that MGL-deficient mice are protected from LPS-induced neuroinflammation by a decrease in inflammatory cytokines observed 6 h after LPS administration [13]. It is possible that the immediate cytokine responses are also lost in *Mgll* ^−/−^ mice. To test this, we examined cytokine responses in *Mgll* ^−/−^ mice. Blood levels of TNFα, IL-1β, and IL-6 were elevated 2 h after systemic LPS administration in both *Mgll* ^−/−^ and WT control mice (Fig 2). A modest attenuation of TNFα levels was noted in *Mgll* ^−/−^ mice (Fig 2*A*) but was unlikely to explain the loss of febrile responses in these mice. Thus, the immediate cytokine responses (~2 h after LPS) that precede the febrile response remained intact in *Mgll* ^−/−^ mice.

### Hypothalamic PGE_2_ is decreased in MGL-deficient mice

**Fig 3:**
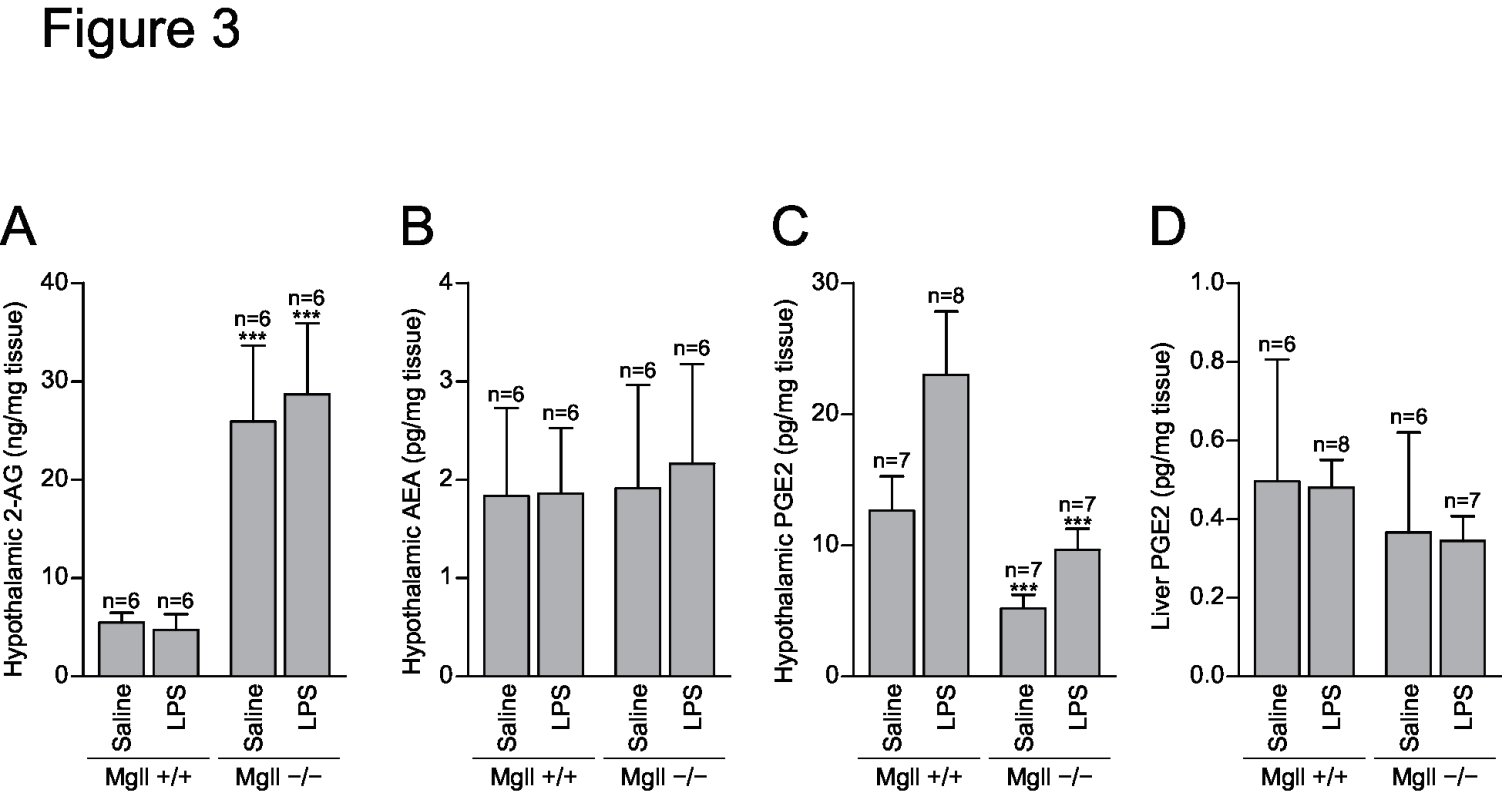
Lipid mediator levels after LPS administration. *Mgll* ^+/+^ and *Mgll* ^−/−^ mice were intraperitoneally injected with 20 μg of LPS or saline (n = 6–8 for each group). Two hours later, hypothalamus and liver tissues were collected. Lipids were extracted and analyzed by liquid chromatography-tandem mass spectrometry. (A) Hypothalamic 2-AG levels. (B) Hypothalamic anandamide levels. (C) Hypothalamic PGE_**2**_ levels. (D) Liver PGE_**2**_ levels. Data are expressed as means ± SD. ***p<0.001 vs. *Mgll* ^+/+^, by Bonferroni post-test after two-way ANOVA.

Next, we performed lipidomics analysis to determine the changes in lipid mediators and related lipids in *Mgll* ^−/−^ mice. Total fatty acid analysis of hypothalamic tissue demonstrated no difference between the genotypes, suggesting that the tissue AA pool is not affected by MGL-deficiency (S1 Fig). Phospholipid profiling analysis reveals no significant difference between the genotypes, suggesting PLA_2_ substrates are unaltered in the *Mgll* ^−/−^ hypothalamus (S2 Fig). Levels of hypothalamic 2-AG levels were substantially increased by the deletion of MGL (Fig 3*A*), while those of anandamide (arachidonoyl ethanolamide, AEA) were not altered (Fig 3*B*). These results confirmed that MGL is the major 2-AG hydrolyzing enzyme in the hypothalamus. Both 2-AG and AEA levels were unaltered by LPS administration in both genotypes (Fig 3*A* and 3*B*), which can be understood either by unchanged 2-AG and AEA production or by possible changes in their metabolic flux, which are undetectable in a snapshot analysis. Hypothalamic PGE_2_ and other several eicosanoids were decreased in *Mgll* ^−/−^ mice to levels no greater than those of saline-treated control WT mice even after LPS administration (Fig 3*C* and S3 Fig). Reports suggest that the liver is an important source of PGE_2_ during LPS-induced fever, as liver Kupffer cells can be activated by LPS [10, 21]. A previous report demonstrated an increase in liver PGE_2_ levels 6 h after LPS administration, which was abolished in MGL-deficient mice [13]. We measured the liver eicosanoids 2 h after LPS administration (Fig 3*D* and S4 Fig). LPS did not increase liver PGE_2_ by 2 h and there were no differences between genotypes, suggesting that MGL-dependent hypothalamic PGE_2_ production is important for febrile response.

### Diurnal core body temperature rhythmicity is normal in MGL-deficient mice

**Fig 4:**
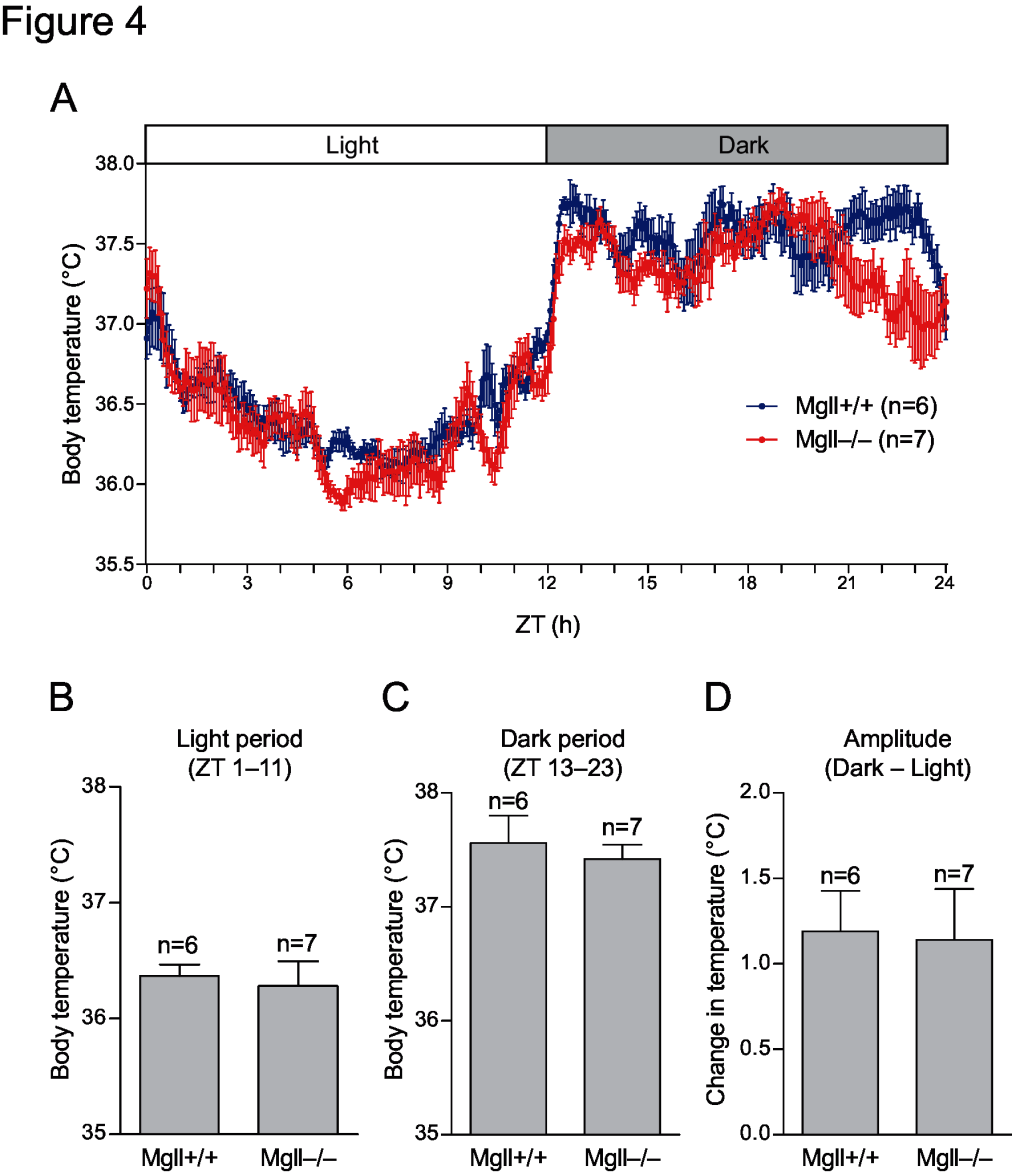
Diurnal core body temperature changes. (A) *Mgll* ^+/+^ (n = 6) and *Mgll* ^−/−^ mice (n = 7) were intra-abdominally implanted with thermo-logger and placed in a thermostat chamber with a light-dark cycle (L:D = 12h:12h). Core body temperatures of 3 consecutive light-dark cycles were averaged for each mice. Data are expressed as means ± SEM, for indicated numbers of mice. No statistically significant differences were found between genotypes (Bonferroni post-test after two-way repeated measures ANOVA). (B-D) Averaged body temperature for light period (ZT 1–10) (B) and dark period (ZT 13–23) (C), and the difference in body temperatures between light and dark periods (D) were calculated using the data shown in (A). No statistically significant differences were found between genotypes (Student’s *t*-test).

To examine if MGL is involved in physiological thermoregulatory mechanisms, diurnal core body temperature changes of *Mgll* ^−/−^ and control WT mice were recorded by intra-abdominal thermo-logger implants. No significant differences in the profile (Fig 4*A*), average core body temperatures in the light and dark periods (Fig 4*B* and 4*C*), and the amplitude of body temperature changes (Fig 4*D*) were found between the genotypes, suggesting that MGL is not involved in the regulation of daily core body temperature rhythms.

## Discussion

Management and medication of fever are clinically important. Non-steroidal anti-inflammatory drugs (NSAIDs) that target COXs, as well as acetaminophen, are widely used for fever treatment and are effective, but safer antipyretics are anticipated since adverse effects are known for these drugs [22]. To this end, detailed understanding of fever mechanism at biochemical levels is essential. In the present study, we demonstrated that MGL-dependent 2-AG degradation is the underlying mechanism for hypothalamic PGE_2_ production in LPS-induced fever. Despite the fact that cPLA_2_α has been regarded as a crucial enzyme for the AA cascade and its involvement has been demonstrated in many inflammatory disease models [9], febrile reaction mediated by hypothalamic PGE_2_-EP3R pathway was an apparent exception; it depends on MGL and does not require cPLA_2_α (Fig 1*A* and 1*B*). Our result suggests that drug(s) that target cPLA_2_α, which is currently not available, would be ineffective against fever. Instead, endocannabinoid metabolism can be a potential target for fever treatment.

Genetic ablation as well as pharmacological inactivation of MGL result in an accumulation of 2-AG *in vivo*, as shown by previous studies [13, 20], and in the present study (Fig 3*A*). It is, therefore, plausible that accumulated 2-AG activates CB1R in the brain to decrease core body temperature (one of the well-known effects of marijuana), which counteracts febrile responses triggered by PGE_2_-EP3R signaling. Although such a hypothesis seems unlikely, as a previous report demonstrated a downregulation of CB1R in MGL-deficient mice due to its persistent activation [20], it is critical to eliminate CB1R signaling to examine the role for MGL in pyretic PGE_2_ production. Our results using compound deficient mice for CB1R and MGL clearly demonstrated MGL-dependent- and CB1R-independent nature of febrile reactions (Fig 1*C*). Moreover, results that ICV injection of PGE_2_ elicited fever in MGL-deficient mice (Fig 1*E*), and that hypothalamic PGE_2_ levels after LPS injection were suppressed by MGL deficiency (Fig 3*C*), further support our concept that MGL mediates pyretic PGE_2_ production in the hypothalamus.

The results for blood levels of TNFα, IL-1β and IL-6 2h after LPS administration demonstrated that pyretic cytokine productions are normal in MGL-deficient mice (Fig 2). A recent report describes that neuroinflammation after LPS administration is suppressed in MGL-deficient mice, with decreased cytokines as well as eicosanoid levels [13]. This discrepancy in cytokine response may be explained by the difference in time points chosen. In the early phase, LPS stimulates a transient increase in blood cytokine levels that reach a peak 1–2 h after LPS administration. This activates hypothalamic PGE_2_-EP3R pathway to cause febrile response. In the later phase, 6–8 h after LPS administration, PGE_2_ (or other MGL-dependent metabolites) on the contrary may enhance cytokine productions in the tissue and exacerbate inflammation. Similar difference was observed for tissue lipid mediators. Previous report describes that LPS-induced elevations in liver prostaglandin E_2_ determined at 6h after LPS administration were blocked by genetic or pharmacological inactivation of MGL [13]. In our experiment, when measured at 2h after injection, LPS did not alter liver PGE_2_ levels in both MGL-deficient and wild-type mice (Fig 3*D*). Meanwhile, levels of TXB_2_, PGD_2_, LTD_4_ and PAF were increased (S4 Fig), demonstrating that LPS do stimulate some lipid mediators at this time point. From the results, we concluded that liver PGE_2_ is not the MGL-dependent factor that mediates febrile responses.

In our experiments using global knockout mice, the cell type(s) producing PGE_2_ in the hypothalamus were not elucidated, thus warranting further studies using conditional knockout mice [23]. A recent study demonstrated that LPS-induced fever depends on PGE_2_ production in the brain endothelial cells [24, 25]. Thus, it is likely that hypothalamic endothelial cells are the candidate cell type also for 2-AG degradation by MGL; however, it is possible that AA released from other cell types leads to endothelial PGE_2_ production by a transcellular mechanism. Observations that COX-2 and mPGES-1 are induced in the endothelial cells during LPS-induced fever [24, 25] indicate that these enzymes seem to be rate-limiting for pyretic PGE_2_ production. It remains elusive whether MGL is also rate-limiting; we did not observe changes in hypothalamic 2-AG levels by LPS (Fig 3*A*), but this does not necessarily mean that MGL activity was unaltered, as concentrations do not reflect possible changes in metabolic flux.

It is of interest that the LPS-induced febrile response was normal in DGLα-deficient mice (Fig 1*D*). We have shown that DGLα expressed in neuronal cells serves as the major 2-AG source in the brain [15] and others have demonstrated MGL as a major 2-AG-degrading enzyme [26], suggesting a functional coupling of DGLα and MGL that regulates 2-AG levels in the brain. However, our results suggest that 2-AG produced independently of DGLα is the precursor for hypothalamic PGE_2_ during fever. Other enzymes with DGL activity (e.g., DGLβ [15]) may be involved; this possibility requires further investigation.

From a physiological point of view, whether PGE_2_ serves as a factor that determines the primary body temperature set point and its diurnal rhythmicity, has been a question. A previous report demonstrates that RANKL-RANK pathway affect diurnal female body temperature changes via PGE_2_-EP3R pathway [27]. In our experiment, MGL-deficient female mice were normothermic, and were similar in both amplitude and rhythmicity of body temperature changes as compared with wild-type mice (Fig 4). The result points that MGL-dependent PGE_2_ production is not involved in such a normal physiology. Thus, hypothalamic PGE_2_ caused by RANKL-RANK signaling in females may involve MGL-independent mechanism(s) in part, which awaits further studies.

## Supporting Information

S1 Fig Total fatty acid profile of hypothalamus. Hypothalamic tissues were collected from *Mgll* ^+/+^ and *Mgll* ^−/−^ mice (n = 6 for each group). Total lipids were extracted and fatty acid methyl esters (FAMEs) were prepared for total fatty acids, including free and esterified fatty acids. FAMEs were quantified using gas chromatography with flame ionization detector (GC-FID). Data are expressed as means ± SD. No statistically significant differences were found between genotypes (Bonferroni post-test after two-way ANOVA).
(PDF)

S2 Fig Phospholipid profile of hypothalamus. Hypothalamic tissues were collected from *Mgll* ^+/+^ and *Mgll* ^−/−^ mice (n = 5 for each group). Phospholipids were extracted from cryomilled samples by methanol and analyzed by liquid chromatography-tandem mass spectrometry. Data are expressed as normalized mean values ± SD, using the total sum of the signals for diradyl-phospholipids or lyso-phospholipids. No statistically significant differences were found between genotypes (Bonferroni post-test after two-way ANOVA). (L)PC, (lyso)phosphatidylcholine; SM, sphingomyelin; (L)PE, (lyso)phosphatidylethanolamine; PS, phosphatidylserine; PI, phosphatidylinositol; LPA, lysophosphatidic acid. Numbers after phospholipid names indicate total carbon and double bond numbers for radyl group(s). ‘o’ and ‘p’ indicate *O*-alkyl and *O*-alkenyl group, respectively.
(PDF)

S3 Fig Hypothalamic lipid mediator levels after LPS administration. *Mgll* ^+/+^ and *Mgll* ^−/−^ mice were intraperitoneally injected with 20 μg of LPS or saline. Two h later, hypothalamic tissues were collected and analyzed for lipid mediator levels by liquid chromatography-tandem mass spectrometry (n = 6–8 for each group). Data are expressed as means ± SD. ***p<0.001 vs. *Mgll* ^+/+^, by Bonferroni post-test after two-way ANOVA.
(PDF)

S4 Fig Liver lipid mediator levels after LPS administration. *Mgll* ^+/+^ and *Mgll* ^−/−^ mice were intraperitoneally injected with 20 μg of LPS or saline. Two h later, liver tissues were collected and analyzed for lipid mediator levels by liquid chromatography-tandem mass spectrometry (n = 2–8 for each group). Data are expressed as means ± SD. *p<0.05 vs. *Mgll* ^+/+^, by Bonferroni post-test after two-way ANOVA.
(PDF)
