# Supplementary material for: Fever Is Mediated by Conversion of Endocannabinoid 2-Arachidonoylglycerol to Prostaglandin E2
Source: PLoS One. 2015 Jul 21;10(7):e0133663. doi: 10.1371/journal.pone.0133663 (PMC4511515; doi:10.1371/journal.pone.0133663)
Supplement: S1 Fig — Hypothalamic tissues were collected from Mgll +/+ and Mgll −/− mice (n = 6 for each group). Total lipids were extracted and fatty acid methyl esters (FAMEs) were prepared for total fatty acids, including free and esterified fatty acids. FAMEs were quantified using gas chromatography with flame ionization detector (GC-FID). Data are expressed as means ± SD. No statistically significant differences were found between genotypes (Bonferroni post-test after two-way ANOVA). (PDF) [file pone.0133663.s001.pdf]

S1 Fig.

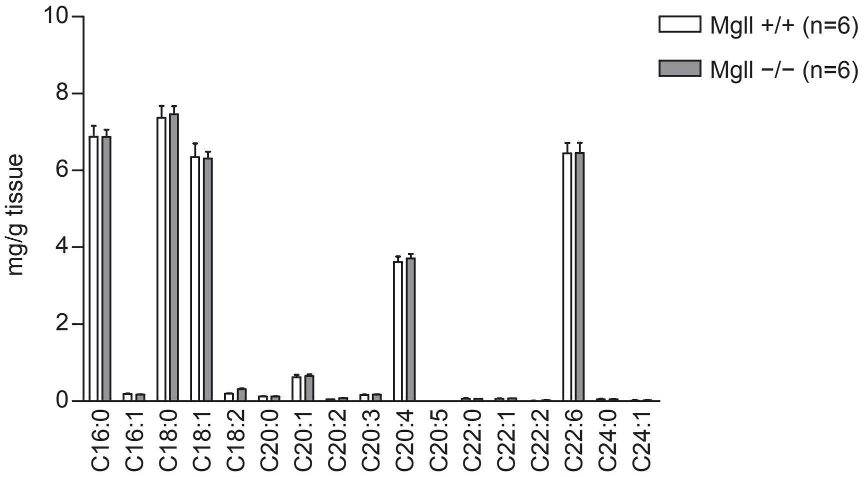

No statistically-significant difference found between WT and KO

(Bonferroni post-test after two-way ANOVA)

Data are expressed as mean ± s.d.
