# Supplementary material for: Fever Is Mediated by Conversion of Endocannabinoid 2-Arachidonoylglycerol to Prostaglandin E2
Source: PLoS One. 2015 Jul 21;10(7):e0133663. doi: 10.1371/journal.pone.0133663 (PMC4511515; doi:10.1371/journal.pone.0133663)
Supplement: S2 Fig — Hypothalamic tissues were collected from Mgll +/+ and Mgll −/− mice (n = 5 for each group). Phospholipids were extracted from cryomilled samples by methanol and analyzed by liquid chromatography-tandem mass spectrometry. Data are expressed as normalized mean values ± SD, using the total sum of the signals for diradyl-phospholipids or lyso-phospholipids. No statistically significant differences were found between genotypes (Bonferroni post-test after two-way ANOVA). (L)PC, (lyso)phosphatidylcholine; SM, sphingomyelin; (L)PE, (lyso)phosphatidylethanolamine; PS, phosphatidylserine; PI, phosphatidylinositol; LPA, lysophosphatidic acid. Numbers after phospholipid names indicate total carbon and double bond numbers for radyl group(s). ‘o’ and ‘p’ indicate O-alkyl and O-alkenyl group, respectively. (PDF) [file pone.0133663.s002.pdf]

S2 Fig.

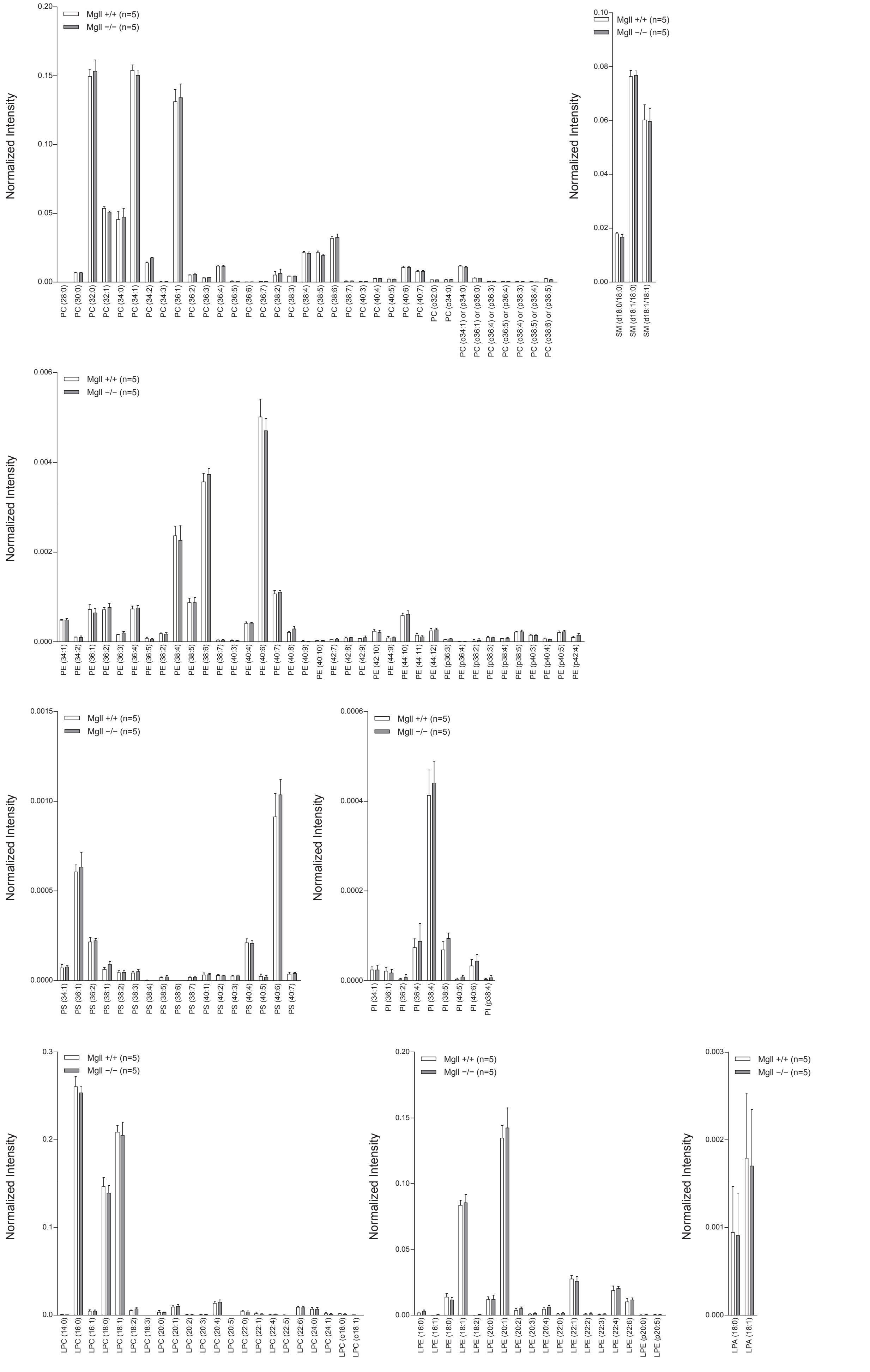

No statistically-significant difference found between WT and KO (Bonferroni post-test after two-way ANOVA)

Data are expressed as mean ± s.d.
