# Supplementary figures and images for: Fever Is Mediated by Conversion of Endocannabinoid 2-Arachidonoylglycerol to Prostaglandin E2
Source: PLoS One. 2015 Jul 21;10(7):e0133663. doi: 10.1371/journal.pone.0133663 (PMC4511515; doi:10.1371/journal.pone.0133663)

S3 Fig.

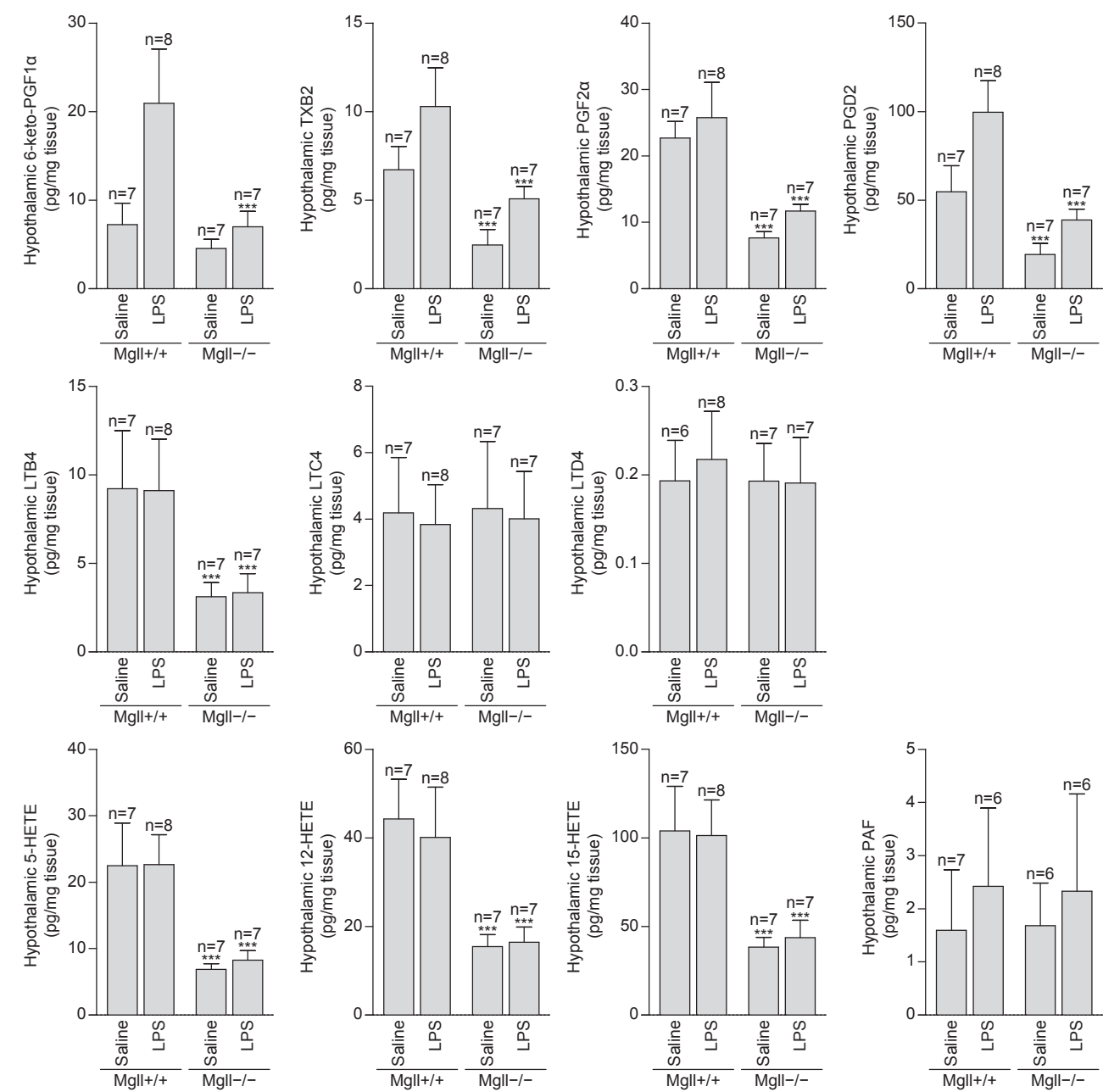

\*\*\*p<0.001, vs. MgII<sup>+/+</sup> (Bonferroni post-test after two-way ANOVA)  
Data are expressed as mean  $\pm$  s.d.

Supplement: S3 Fig — Mgll +/+ and Mgll −/− mice were intraperitoneally injected with 20 μg of LPS or saline. Two h later, hypothalamic tissues were collected and analyzed for lipid mediator levels by liquid chromatography-tandem mass spectrometry (n = 6–8 for each group). Data are expressed as means ± SD. ***p<0.001 vs. Mgll +/+, by Bonferroni post-test after two-way ANOVA. (PDF) [file pone.0133663.s003.pdf]

S4 Fig.

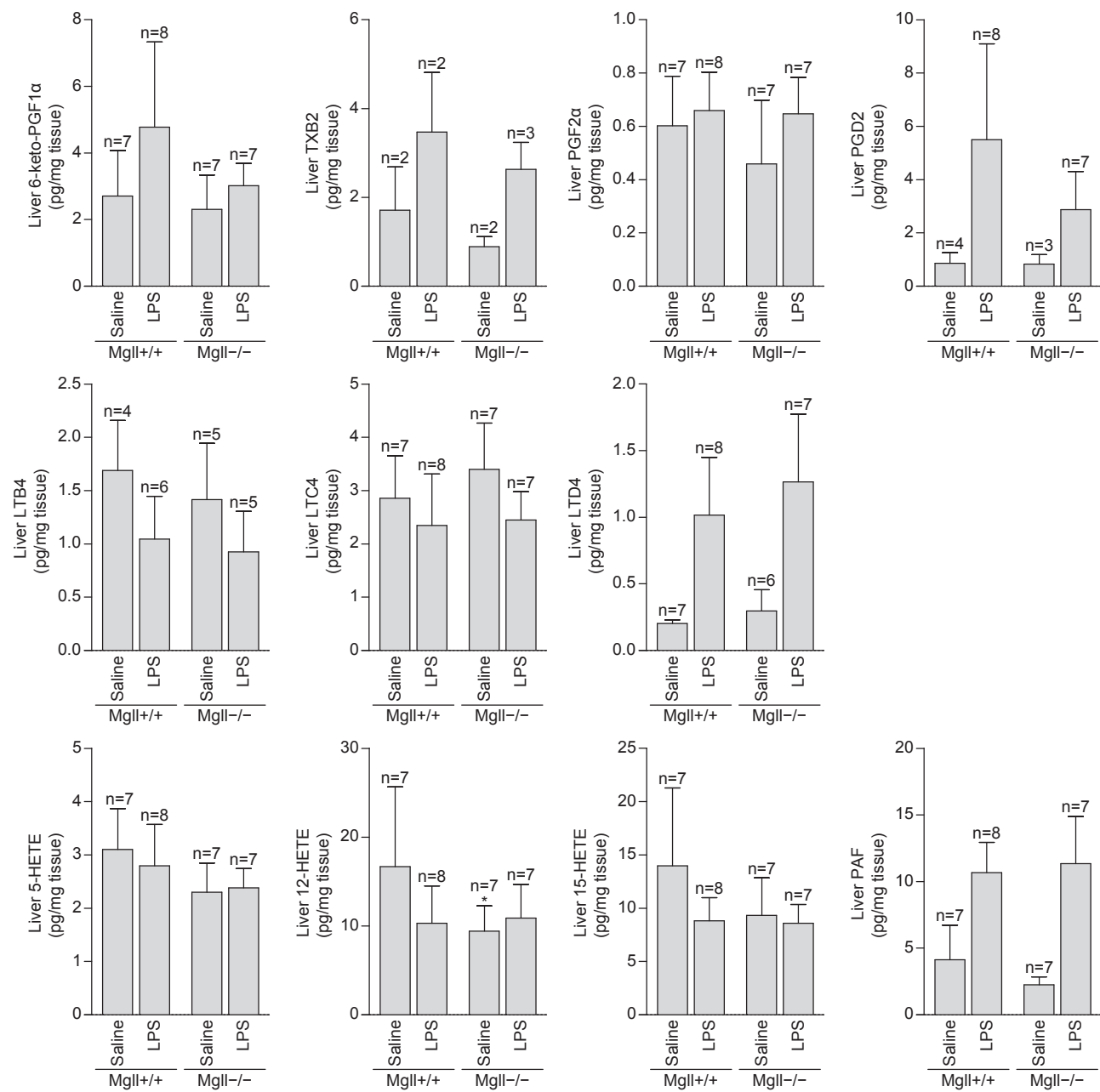

\*p<0.05, vs. MgII<sup>+/+</sup> (Bonferroni post-test after two-way ANOVA)  
Data are expressed as mean  $\pm$  s.d.

Supplement: S4 Fig — Mgll +/+ and Mgll −/− mice were intraperitoneally injected with 20 μg of LPS or saline. Two h later, liver tissues were collected and analyzed for lipid mediator levels by liquid chromatography-tandem mass spectrometry (n = 2–8 for each group). Data are expressed as means ± SD. *p<0.05 vs. Mgll +/+, by Bonferroni post-test after two-way ANOVA. (PDF) [file pone.0133663.s004.pdf]
